# Exogenous Carbon Monoxide Produces Rapid Antidepressant- and Anxiolytic-Like Effects

**DOI:** 10.3389/fphar.2021.757417

**Published:** 2021-11-18

**Authors:** Yixiao Luo, Rafi Ullah, Jinfeng Wang, Yuru Du, Shihao Huang, Li Meng, Yuan Gao, Miao Gong, Ewa Galaj, Xi Yin, Haishui Shi

**Affiliations:** ^1^ Key Laboratory of Molecular Epidemiology of Hunan Province, School of Medicine, Hunan Normal University, Changsha, China; ^2^ Neuroscience Research Center, Institute of Medical and Health Science of HeBMU, Hebei Medical University, Shijiazhuang, China; ^3^ Department of Obstetrics and Gynecology, The No.1 Hospital of Yongnian District Handan City, Handan, China; ^4^ Hebei Key Laboratory of Neurophysiology, Hebei Medical University, Shijiazhuang, China; ^5^ Neuroscience Program, Department of Psychological and Brain Sciences, Colgate University, Hamilton, NY, United States; ^6^ Department of Functional Region of Diagnosis, Fourth Hospital of Hebei Medical University, Shijiazhuang, China

**Keywords:** carbon monoxide, heme oxygenase-1, depression, anxiety, inflammation

## Abstract

Carbon monoxide (CO), a byproduct of heme catalyzed by heme oxygenase (HO), has been reported to exert antioxidant and anti-inflammatory actions, and to produce significant neuroprotective effects. The potential effects of CO and even HO on depressive-like behaviors are still poorly understood. Utilizing several approaches including adeno-associated virus (AAV)-mediated overexpression of HO-1, systemic CO-releasing molecules (CO-RMs), CO-rich saline or CO gas treatment procedures in combination with hydrogen peroxide (H_2_O_2_)-induced PC12 cell injury model, and lipopolysaccharide (LPS)-induced depression mouse model, the present study aimed to investigate the potential antidepressant- and anxiolytic-like effects of endogenous and exogenous CO administration *in vivo* and *in vitro*. The results of *in vitro* experiments showed that both CO-RM-3 and CO-RM-A1 pretreatment blocked H_2_O_2_-induced cellular injuries by increasing cell survival and decreasing cell apoptosis and necrosis. Similar to the effects of CO-RM-3 and CO-RM-A1 pretreatment, AAV-mediated HO-1 overexpression in the dorsal hippocampus produced significant antidepressant-like activities in mice under normal conditions. Further investigation showed that the CO gas treatment significantly blocked LPS-induced depressive- and anxiety-like behaviors in mice. Taken together, our results suggest that the activation of HO-1 and/or exogenous CO administration produces protective effects and exerts antidepressant- and anxiolytic-like effects. These data uncover a novel function of the HO-1/CO system that appears to be a promising therapeutic target for the treatment of depression and anxiety.

## 1 Introduction

Major depressive disorder (MDD) is one of the most common mental illnesses worldwide, which can cause suicide, disability, problem behaviors, and limitations in day-to-day activities (Trivedi et al., 2006; Kaufman, 2018; Domschke and Gottschalk, 2019). Therefore, the search for rapid and effective antidepressants is of high interest and paramount importance. Chronic neural and peripheral inflammation are associated with depression-related symptoms and reversed by antidepressant treatments in MDD patients (Dowlati et al., 2010; Martinowich et al., 2013; Hodes et al., 2015; Goldsmith et al., 2016; Liu et al., 2017c). In rodents, both chronic mild stress (CMS) exposure and systemic lipopolysaccharide (LPS) administration can induce inflammatory responses, increase significant depressive- and anxiety-like behaviors, and also cause memory impairments (Yin et al., 2019), which can be blocked by some antioxidant and anti-inflammatory agents such as flavonoid and sulfur dioxide (SO_2_) (Hritcu et al., 2017; Shi et al., 2020). Further attention has been given to oxidative stress injury and excessive inflammation as targets in the development of therapeutic strategies for depression.

Carbon monoxide (CO), produced by incomplete combustion of organic compounds, has been the focus of toxicology research as it can cause acute poisoning. However, carbon monoxide is also formed endogenously in the body as a byproduct of heme degradation, catalyzed by the action of heme oxygenase (HO) enzymes. Two types of HO isoforms are known in mammals: stress-inducible HO-1 and constitutively expressed HO-2 (Maines, 1997). HO-1 generates CO by catalyzing the cleavage of heme and produces anti-inflammatory and antioxidant effects *in vivo* and *in vitro* (Otterbein et al., 2000; Jazwa and Cuadrado, 2010; Chen, 2014; Zhang et al., 2014). In addition, HO-1 was identified as a potential therapeutic target for the treatment of several neurological diseases such as ischemia/reperfusion injury, subarachnoid hemorrhage, and neurodegenerative diseases (Maines, 2002; Kotsch et al., 2007; Jazwa and Cuadrado, 2010; LeBlanc et al., 2016; Chen-Roetling and Regan, 2017). Interestingly, exogenous CO administration can also exhibit significant protective effects from a broad range of physiological and pathological processes (Motterlini and Otterbein, 2010; Choi et al., 2016). Recently, several studies suggested that CO inhalation could prevent anesthesia-induced neurotoxicity, enhance survival, and promote neurogenesis after traumatic brain injury (TBI) (Queiroga et al., 2015; Choi et al., 2016; Yan et al., 2016). The CO-releasing molecules (CO-RMs) are a group of chemical compounds that can directly control the release of CO in tissues or organs (Motterlini et al., 2002; Clark et al., 2003; Motterlini et al., 2005). CO-RM treatment can also produce neuroprotective effects in inflammation-related brain diseases, such as ischemia–reperfusion injury (IRI), hemorrhagic stroke, and traumatic brain injury (Taskiran et al., 2003; Wang and Dore, 2007; Tsoyi et al., 2009; Wang et al., 2011; Yabluchanskiy et al., 2012; Lin et al., 2017; Ulbrich et al., 2017; Wilson et al., 2017). Recently, HYCO-3, a dual CO releaser/Nrf2 coactivator, has been reported to reduce tissue inflammation in mice challenged with LPS, highlighting the potentiality of HO-1/CO for the treatment of inflammation-related brain diseases (Motterlini et al., 2019). However, whether CO has a direct effect on depressive- and anxiety-like behaviors and its mechanisms of action are still elusive.

In the present study, the role of HO-1 on depression and anxiety was investigated by using adeno-associated virus (AAV)-mediating overexpression in the dorsal hippocampus region of mice. Furthermore, two CO-RMs, CO-RM-3 and CO-RM-A1, were selected to investigate the protective effects of CO in H_2_O_2_-induced PC12 cell injury (Clark et al., 2003; Motterlini et al., 2005). The antidepressant- and anxiolytic-like effects of systemic exogenous CO application (including CO-RM-A1, CO-RM-3, CO gas, or CO-rich saline injection) were investigated by using several gold-standard behavioral tests in mice under normal conditions or exposure to LPS.

## 2 Materials and Methods

### 2.1 Animals

Eight-week-old male Kunming mice (initially weighed 20–22 g on arrival) were purchased from Beijing Vital River Laboratory Animal Technology Co., Ltd. (Beijing, China). Mice were housed in groups of five in 320 × 215 × 170 mm cages at a constant temperature (23 ± 2°C) with a 12 h light/dark cycle (lights off from 8:00 a.m. to 8:00 p.m.), with free access to food and water. All behavioral experiments were conducted during the dark period and according to the guidelines of the National Institutes of Health Guide for the Care and Use of Laboratory Animals. The present study was reviewed and approved by the Laboratory Animal Ethical and Welfare Committee of Hebei Medical University.

### 2.2 Agents

LPS, CO-RM-3, and CO-RM-A1 were purchased from Sigma (Sigma-Aldrich, St. Louis, MO, United States). CO gas (purify >99.99%) was purchased from Wuhan Newradar Trade Company Limited (Wuhan, China). The dosages of CO-RM and CO-rich saline were prepared based on previous studies (Wang et al., 2018b; Chu et al., 2019; Li et al., 2019). The FITC Annexin V apoptosis Detection Kit I was purchased from BD Pharmingen^TM^ (BD Biosciences, China). HO-1 primary antibody (ab13243, United States) and β-actin primary antibody (ab8227, United States) were purchased from Abcam. The CellTiter96 AQueous One Solution Cell Proliferation Assay Kit was purchased from Promega Corporation (CAT3580, United States).

### 2.3 Cell Culture and Treatment

PC12 cells (from Prof. Di Wen, Hebei Medical University) were cultured in the high-glucose Dulbecco’s modified Eagle’s medium (DMEM) with 10% fetal bovine serum (FBS) at 37°C in a humidified incubator containing 5% of CO_2_. According to our previous study (Wang et al., 2019), for the drug treatment, the cells were seeded onto a 96-well culture plate at 5 × 10^7^ cells/well until full confluence (70–80%). After the treatment with CO-RM or saline for 1h, cells were incubated with H_2_O_2_ (200 µΜ) for another 24 h in the culture medium.

### 2.4 Production of Adeno-Associated Virus and Stereotaxic Viral Injection

The control plasmid (GFP-OE), recombinant HO-1 (NM_010442) overexpression plasmid (CMV bGlobin-MCS-EGFP-3FLAG-WPRE-hGH polyA, HO-1-GFP OE), and packaged AAV were purchased from Shanghai GeneChem Co. Ltd. (Shanghai, China). Mice were anesthetized with sodium pentobarbital (150 mg/kg, i.p.) and placed in a stereotaxic frame. Mice (*n* = 7–9) were infused bilaterally with 0.3 μl of purified and concentrated AAV (1.8E+13 v.g/ml) into the dorsal hippocampus region [anterior–posterior (Goldsmith et al., 2016): −2.1 mm, medial lateral (Fan et al., 2017): ±1.4 mm, dorsal ventral (DV): −1.5 mm] using an electric microinjection pump (RWD Life Science Co., Ltd., China) at a rate of 150 nl/min. Behavioral tests were performed 21 days after AAV microinfusion.

### 2.5 MTS Assay

The MTS assay was conducted according to the kit’s protocol (Wang et al., 2019). Briefly, PC12 cells (5,000 cells/well) were seeded into 96-well microtiter plates. After incubation with 10–400 μM of CO-RM-3 or CO-RM-A1 for 1 h, the cells were co-incubated with 200 μM of H_2_O_2_ for another 24 h. Subsequently, 20 μl of MTS solution was added to each well, and the plates were incubated for 3 h at 37°C. Absorbance was measured at 470 nm and used to calculate the relative ratio of cell viability.

### 2.6 Flow Cytometry Analysis

The FITC Annexin V Apoptosis Detection Kit (BD Pharmingen^TM^, United States) was used for detecting the apoptosis (Wang et al., 2019). Annexin V binding and PI (propidium iodide) staining were determined by flow cytometry (EasycyteTm mini system). The cells were treated with 200 μM of CO-RM-3 for 1 h before incubation with 200 μM of H_2_O_2_ for 24 h, and washed with ice-cold PBS, and then double-stained for 20 min with FITC-coupled Annexin V protein and PI. Flow cytometry was carried out with a 488-nm laser coupled to a cell sorter (FACSCaliburTM; BD Biosciences). Cells stained with both PI and Annexin V were considered necrotic, and those stained only with Annexin V were considered apoptotic.

### 2.7 Immunofluorescence Assay

On the last day of behavioral testing, mice in each group were randomly selected and were anesthetized with sodium pentobarbital (150 mg/kg, i.p.), and slowly perfused with 4% paraformaldehyde (PFA). Their brains were removed and post-fixed with the same fixative overnight at 4°C followed by graded dehydration (Han et al., 2019). Paraffin-embedded brain slices (20 μm) were incubated with the primary polyclonal rabbit anti–HO-1 (1:500) antibody followed by the fluorescent-conjugated secondary antibody (goat anti-rabbit IgG, 1:200; Sigma-Aldrich). Images were acquired with the use of a scanning laser confocal microscope (LSM780, Carl Zeiss, Germany).

### 2.8 Western Blot Analysis

To detect the expression of HO-1 in the dorsal hippocampus region of mice (*n* = 4), on the last day of behavioral testing, mice were anesthetized with sodium pentobarbital (150 mg/kg, i.p.) and were decapitated for collection of dorsal hippocampus tissue. Protein samples were prepared and analyzed by Western blot. The polyclonal rabbit anti–HO-1 (1:1,000) and anti–β-actin (1:8,000) were used in the present study. Protein band densities were quantified using ImageJ software and were normalized to β-actin (Shi et al., 2012).

### 2.9 Pretreatment With CO

CO-RM or saline was administered before H_2_O_2_/saline treatment (in Exp. 2; *n* = 6–9 per group) or prior to behavioral testing (in Exp. 3; *n* = 8–9 per group). CO gas or saline was administered prior to behavioral testing in the absence (Exp. 4; *n* = 8–9 per group) or presence (Exp. 5; *n* = 8–9 per group) of LPS-induced depression. Mice were randomly assigned to experimental conditions.

### 2.10 Lipopolysaccharide-Induced Depression Mice Model

Based on previous studies, LPS was diluted in saline at a constant dose of 0.83 mg/kg and administered intraperitoneally for 3 days. Twenty-four hours after LPS or saline administration in Exp. 5, behavioral tests were conducted in the presence or absence of CO pretreatment. Mice were randomly assigned to one of the following conditions: sal+sal; sal+LPS; saline, 0.1 ml CO+LPS, or 0.3 ml CO+LPS condition (*n* = 8–9 per group).

### 2.11 Behavioral Tests

#### 2.11.1 Sucrose Preference Test

The sucrose preference test (SPT) was performed to measure anhedonia of mice subjected to chemogenetic manipulation (Exp. 1) or CO gas pretreatment in the absence (Exp. 4) or presence (Exp. 5) of LPS-induced administration. During the habituation session, two bottles of 1% sucrose solution (w/v) were provided in each cage for 48 h. Prior to the test session, mice were deprived of water for 24 h and then individually housed before being exposed to two bottles (one bottle was filled with 1% sucrose solution, and the other was filled with water) for 24 h. Sucrose consumption and water consumption during the test session were measured as sucrose preference (Shi et al.) = sucrose consumption/(sucrose consumption + water consumption) ×100. The position of sucrose and water bottles was counterbalanced between cages to control for preference bias.

#### 2.11.2 Forced Swimming Test

The forced swimming test (FST) was performed to test the despair behavior of mice (Wu et al., 2016). Mice were placed in a plastic cylinder (diameter 12 cm, tall 30 cm) filled up to 15 cm with 23–25°C water. Latency to immobility during the first 2 min and immobility time during the last 4 min were measured. Immobility was defined as a lack of movement, except motions required to maintain the animal’s head above the water. Floating time was measured by experimenters blinded for experimental conditions.

#### 2.11.3 Novelty-Suppressed Feeding Test

The novelty-suppressed feeding (NSF) test was performed to detect anxiety-like behaviors of mice (Zhu et al., 2012; Wu et al., 2016). After 24 h food deprivation, the mice were transferred to a testing room. The subjects were tested in an open-field box (40 cm × 40 cm × 40 cm). A small piece of mouse chow was placed in the center of the box. Each mouse was placed in the corner of the testing arena, and the time to the first feeding episode was recorded as “latency to feeding.” Immediately after the mouse began to eat the chow, the tested animal was removed and placed alone in a clean cage with a weighed piece of chow for 10 min. The total food intake of each mouse in the cage was assessed to reflect the appetite of mice.

#### 2.11.4 Open Field Test

The open field test (OFT) was conducted to detect the anxiety-like behavior of mice based on previous reports (Wu et al., 2016; Gong et al., 2017). The Plexiglas arena (40 cm × 40 cm × 40 cm) was divided into the center zone (20 cm × 20 cm) and periphery zone. Each mouse was put in the center area at the beginning and allowed to explore freely for 5 min. The time spent in the center and total distance traveled were recorded automatically by a computer-based video-tracking system and analyzed by software (SMART v3.0.02).

#### 2.11.5 Tail Suspension Test

According to our previous report (Gong et al., 2017), for the tail suspension test (TST), mice were suspended 50 cm above the floor using an adhesive tape placed 1 cm from the tip of the tail. The immobility latency during the first 2 min and immobility time during the last 4 min were measured. Immobility was defined as the immobility of the limb or body when a mouse was hung passively. During the test, mice were separated from each other to prevent possible visual and acoustical interplay. All data were recorded by individuals blinded for this experiment. Observers were blinded to the treatment groups.

### 2.12 Data Analysis

The sample size per experimental condition is that each experiment was determined based on a power analysis indicating that *n* = 7–9 is sufficient to achieve significant results. All data were expressed as mean ± SEM. One-way analyses of variance (ANOVAs) (Basuroy et al.) were used for all data analyses followed by Dunnett’s *post hoc* tests for multiple group comparisons through SPSS 21.0 statistical software. We ensured that the assumption of parametric statistics was met before using ANOVAs. Levene’s tests of homogeneity indicated equal variance across groups in all experiments (*p* > 0.05) and the Shapiro–Wilk test confirmed that the data were normally distributed in all experiments (*p* > 0.05). The values of *p* < 0.05 were considered statistically significant.

## 3 Results

### 3.1 Dorsal Hippocampal Overexpression of HO-1 Produced Antidepressant Activities

**FIGURE 1:**
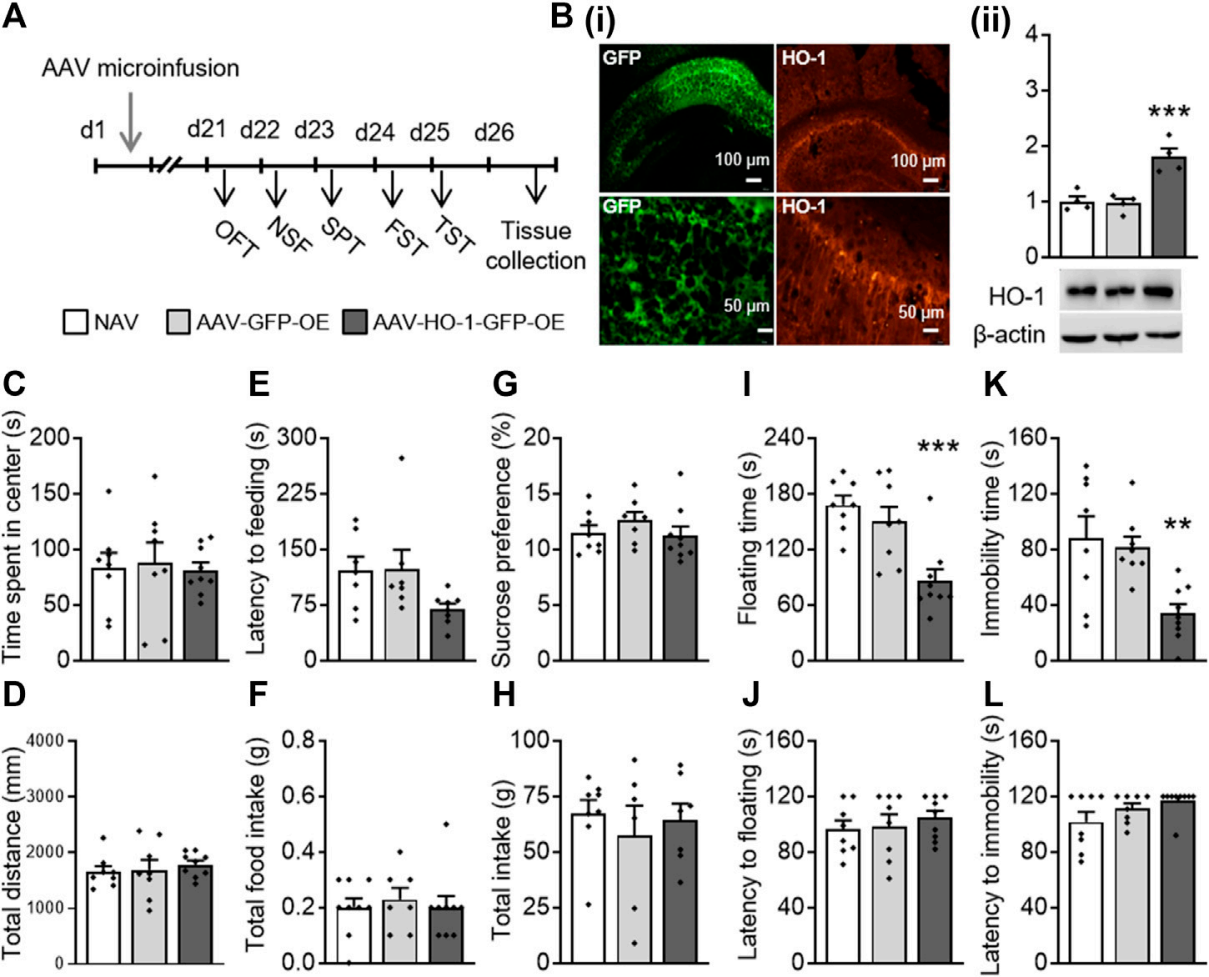
Dorsal hippocampal HO-1 overexpression produced antidepressant-like effects. **(A)** AAV-HO-1 virus was infused bilaterally into the dorsal hippocampus of mice before behavioral testing. **(B)** HO-1 protein levels were significantly increased in the dorsal hippocampus area, as imitated by immunofluorescence staining **(i)** and Western blot **(ii)**. Time spent in the central zone **(C)** and total distance **(D)** were similar across all groups in the OFT. HO-1 overexpression has no effect on latency to feeding **(E)** and total food intake **(F)** in the NSF test. No change occurred in sucrose preference **(G)** and total intake **(H)** after administration of AAV-HO-1 in the SPT. HO-1 overexpression significantly decreased the floating time **(I)** in the FST and immobility time **(K)** but not floating latency **(J)** and immobility latency in the TST **(L)**. Dorsal hippocampal AAV-GFP infusion had no effects on these behaviors. The data are expressed as mean ± SEM and were analyzed with separate one-way ANOVAs. **p < 0.01, ***p < 0.001 versus naive group. NAV, naive; AAV, adeno-associated virus; HO-1, heme oxygenase-1; OFT, open field test; NSF, novelty-suppressed feeding; SPT, sucrose preference test; FST, forced swimming test; TST, tail suspension test (*n* = 4 per group for WB analysis, *n* = 7–9 per group for behavioral tests).

First, we investigated the effect of HO-1 overexpression in the dorsal hippocampus on depressive- and anxiety-like behaviors. The AAV-HO-1 or AAV-GFP control virus was constructed and infused bilaterally into the dorsal hippocampus region of mice (Figure 1A). Immunofluorescence staining results showed that both GFP (Yabluchanskiy et al., 2012) and HO-1 (Sindermann et al., 2021) were expressed in the mouse dorsal hippocampus region (Figure 1Bi). A one-way ANOVA revealed that there were significant differences in the HO-1 protein level (F_(2,11)_ = 18.419, *p* < 0.001, Figure 1Bi) between groups. A *post hoc* analysis revealed that HO-1 protein levels were significantly increased in the dorsal hippocampus area of mice microinfused with AAV-HO-1 virus as compared to those of NAV (naïve) (*p* = 0.001) or AAV-GFP–treated groups (*p* < 0.001).

We then conducted a series of behavioral testing to determine whether chemogenetic overexpression of HO-1 produces antidepressant-like responses. In the OFT, no significant difference was observed in time spent in the center (F_(2,24)_ = 0.063, *p* = 0.939; Figure 1C) and in the total distance (F_(2,24)_ = 0.379, *p* = 0.689; Figure 1D) traveled by naïve, AAV-GFP-OE, and AAV-HO-1-GFP-OE groups. In the NSF, HO-1 overexpression had no significant effect on latency to feeding (F_(2,24)_ = 1.640, *p* = 0.215; Figure 1E) and on total food intake (F_(2,24)_ = 0.286, *p* = 0.754; Figure 1F). In the SPT, HO-1 overexpression within the dorsal hippocampus showed no significant effect on sucrose preference (F_(2,24)_ = 0.481, *p* = 0.624; Figure 1G) and total water intake (F_(2,24)_ = 0.858, *p* = 0.437; Figure 1H). However, significant differences between groups were revealed in the FST and TST. One-way ANOVAs on floating time in the FST (F_(2,24)_ = 11.237, *p* < 0.001, Figure 1I) and immobility time in the TST (F_(2,24)_ = 7.715, *p* = 0.003, Figure 1K) revealed significant differences between groups. A *post hoc* analysis showed that overexpression of HO-1 within the dorsal hippocampus led to significant antidepressant activities, as indicated by decreased floating time (*p* < 0.001) and immobility time (*p* = 0.003), but did not affect the latency to floating (Figure 1J) and latency to immobility (Figure 1L), as compared to mice receiving an AAV-vector control injection and naïve mice. These results indicated that the dorsal hippocampus HO-1 overexpression induced antidepressant-like activities in mice that were evident in the FST and TST.

### 3.2 CO-RM Pretreatment Blocked H_2_O_2_-Induced PC12 Cell Injury

**FIGURE 2:**
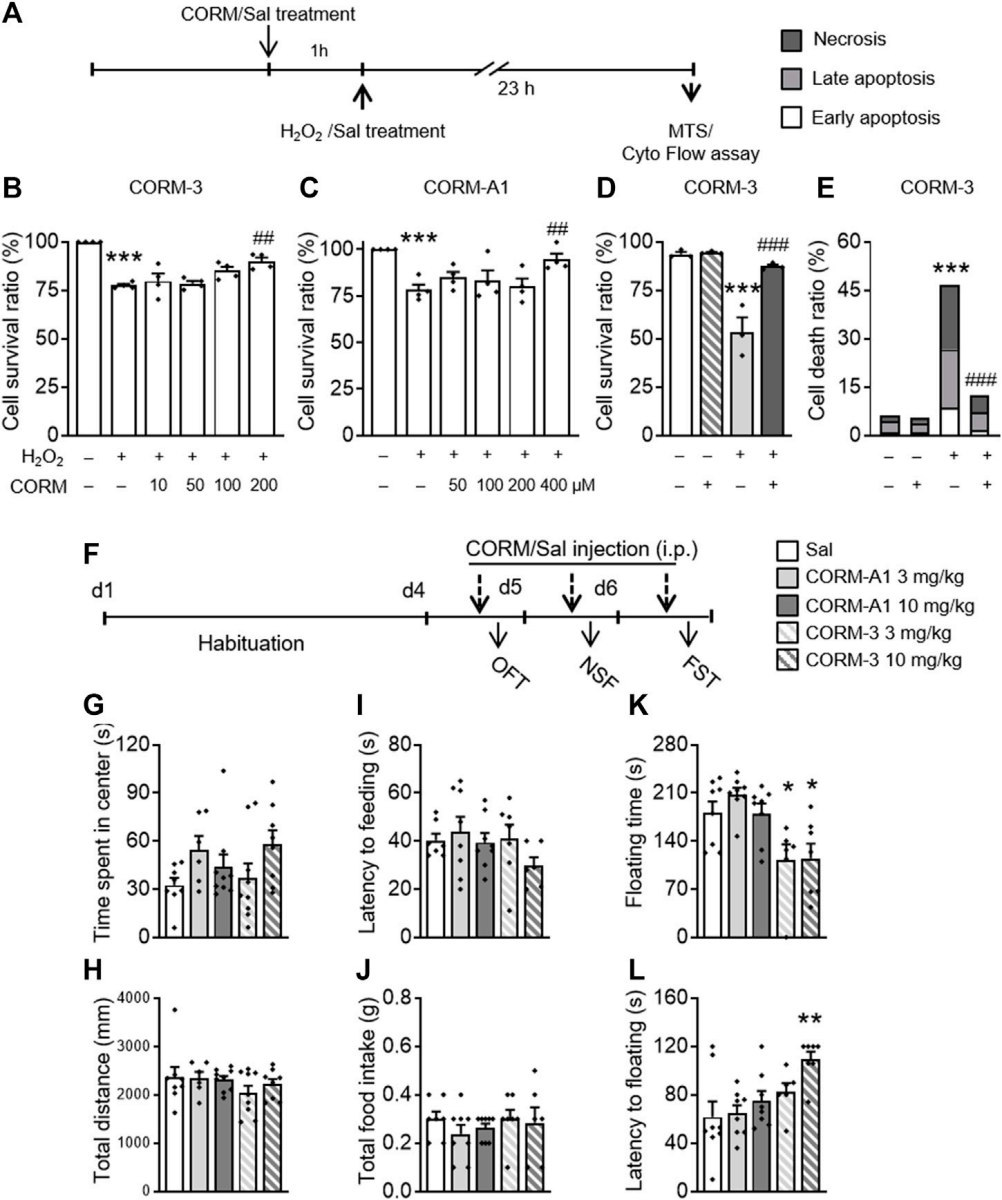
CO-RM pretreatment exerted protective effects *in vitro* and *in vivo*. **(A)** CO-RM donor/saline was administered 1 h before H_2_O_2_/saline treatment; MTS or flow cytometry assay was conducted 24 h later. 100, 200 μM CO-RM-3 **(B)**, and 400 μM CO-RM-A1 **(C)** preconditioning significantly blocked H_2_O_2_-induced PC12 cell injury in the MTS assay. Protective effects of CO-RM-3 pretreatment **(D)** would be associated with its inhibitory role on H_2_O_2_-induced necrosis and apoptosis **(E)** detected by the flow cytometry assay. **(F)** CO-RM-A1 (3, 10 mg/kg, i.p.) or CO-RM-3 (3, 10 mg/kg, i.p.) was administered daily 30 min prior to behavioral testing. CO-RM-A1 and CO-RM-3 administration had no effect on time spent in the central zone **(G)** and total distance **(H)** during the OFT. CO-RM-A1 and CO-RM-3 administration did not affect the latency to feeding **(I)** and total intake of food **(J)** in the NSF test. CO-RM-3 (3, 10 mg/kg) significantly reduced floating time **(K)** in the FST. CO-RM-3 treatment at 10 mg/kg dose significantly increased the floating latency **(L)** in the FST. The data are expressed as mean ± SEM and were analyzed with separate one-way ANOVAs. ***p < 0.001 as compared to the control group; ^#^ *p* < 0.05, ^##^ *p* < 0.01, ^###^ *p* < 0.001 as compared to the H_2_O_2_-treated group; *p < 0.05, **p < 0.01 as compared to the Sal group; Sal, saline. CO-RM, carbon monoxide–releasing molecule; OFT, open field test; NSF, novelty-suppressed feeding; FST, forced swimming test (*n* = 4 per group for cellular analysis, *n* = 6–9 per group for behavioral tests).

To determine the potential effects of CO, two commonly used CO-RMs, CO-RM-3 and CO-RM-A1, were used in the H_2_O_2_-induced cell injury experiment. Four concentrations of each CO-RM were used for subsequent experiments: CO-RM-3 (10, 50, 100, and 200 μM) and CO-RM-A1 (50, 100, 200, and 400 μM). One-way ANOVAs of the MTS test showed there were significant differences in the survival rates (F_(5,24)_ = 20.765, *p* < 0.001, Figure 2B; F_(5,24)_ = 7.795, *p* < 0.001, Figure 2C) between groups. A *post hoc* analysis revealed that H_2_O_2_ treatment significantly reduced the cell survival ratio (*p* < 0.001, Figure 2B; *p* < 0.001, Figure 2C), which was blocked by 200 μM CO-RM-3 (*p* = 0.001) and 400 μM CO-RM-A1 (*p* = 0.004) pretreatments for 1h, indicating a dose-dependent protection of CO-RMs against H_2_O_2_-induced PC12 cell injury. One-way ANOVAs on the flow cytometry data revealed significant differences in the cell survival ratio (F_(3,11)_ = 25.383, *p* < 0.001, Figure 2D) and cell death ratio (F_(3,11)_ = 25.242, *p* < 0.001, Figure 2E) between groups. The *post hoc* analysis showed that the H_2_O_2_ treatment significantly reduced the cell survival ratio (*p* < 0.001) and increased the cell death ratio (*p* < 0.001), which was all prevented by pretreatment with 200 μM CO-RM-3 (*p* = 0.001; *p* = 0.001). These results confirmed that CO-RMs protect PC12 cells from H_2_O_2_-induced apoptosis, exerting significant protective effects in a dose-dependent manner.

### 3.3 CO-Releasing Molecule Treatment Exerted Rapid Antidepressant-Like Activities

In the next experiment, to determine whether CO produces antidepressant- and anxiolytic-like effects in mice, we conducted a battery of behavioral tests after mice were repeatedly treated with different doses of CO-RM-A1 and CO-RM-3. After a 3-day adaptation period, mice received injections of CO-RM-A1 (3, 10 mg/kg, i.p) or CO-RM-3 (3, 10 mg/kg, i.p) interperitoneally 30 min before behavioral tests (Figure 2F). CO-RM-A1 and CO-RM-3 administration had no effect on time spent in the central zone (F_(4,36)_ = 0.905, *p* = 0.472; Figure 2G) and total distance traveled (F_(4,36)_ = 1.854, *p* = 0.14; Figure 2H) in the OFT. CO-RM-A1 and CO-RM-3 administration did not affect the latency to feeding (F_(4,36)_ = 1.538, *p* = 0.212; Figure 2I) and total intake of food (F_(4,36)_ = 0.893, *p* = 0.478; Figure 2J) in the NSF test. However, significant differences between groups were observed in the FST.

One-way ANOVAs on floating time (F_(4,36)_ = 6.272, *p* = 0.001, Figure 2K) and latency to floating (F_(4,36)_ = 4.492, *p* = 0.005, Figure 2L) revealed significant differences between groups. A *post hoc* analysis showed that 3 mg/kg (*p* = 0.031) and 10 mg/kg (*p* = 0.031) CO-RM-3 treatment decreased floating time, while 10 mg/kg CO-RM-3 increased latency to floating (*p* = 0.002) of mice as compared to the vehicle group. Thus, these data suggest that CO-RMs produce antidepressant-like effects.

### 3.4 CO-Rich Saline Treatment Exerted Significant Antidepressant- and Anxiolytic-Like Activities

**FIGURE 3:**
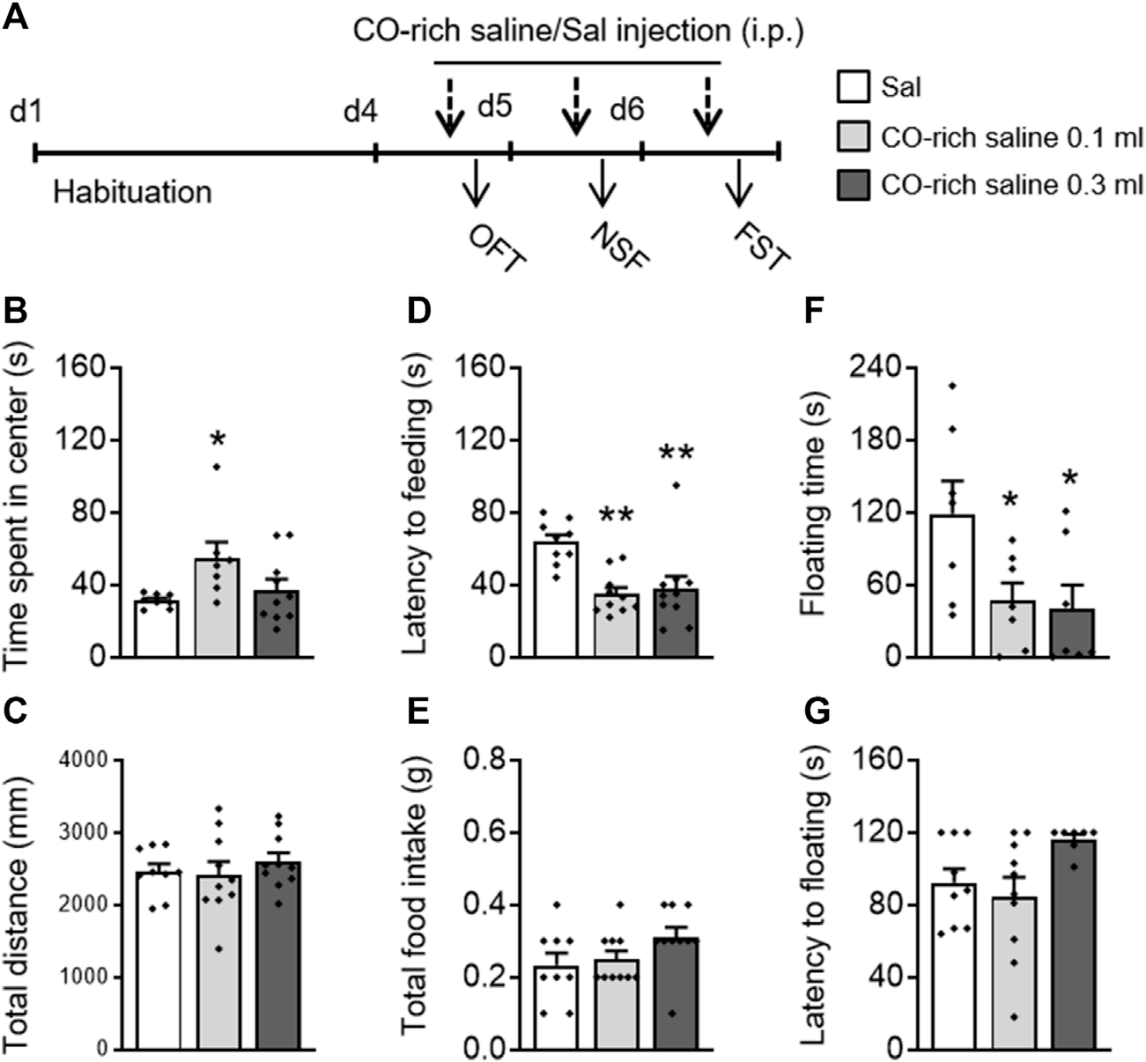
Systemic CO-rich saline pretreatment exerted rapid antidepressant- and anxiolytic-like effects in mice. **(A)** Saline (0.3 ml, i.p.) or CO-rich saline (0.1, 0.3 ml, i.p.) was administered daily 30 min before each behavioral test. CO-rich saline (0.1 ml) treatment significantly increased time spent in the central zone **(B)** without effecting locomotion activity, demonstrated by the total distance traveled **(C)** in the OFT. CO-rich saline treatment significantly decreased latency to feeding **(D)** but not total food intake **(E)** in the NSF test. In the FST, floating time **(F)** was decreased by CO-rich saline but not latency to floating **(G)**. The data are expressed as mean ± SEM and were analyzed with separate one-way ANOVAs. *p < 0.05, **p < 0.01, as compared to the saline-treated group; Sal, saline. OFT, open field test; NSF, novelty-suppressed feeding; FST, forced swimming test (*n* = 8–9 per group).

CO-rich saline (0.1, 0.3 ml, i.p.) was used to explore whether direct administration of CO has antidepressant- and anxiolytic-like effects (Figure 3A). A one-way ANOVA on the OFT data revealed that CO-rich saline had an effect on time spent in the center (F_(2,24)_ = 3.463, *p* = 0.049; Figure 3B) without influencing the total movement distance (F_(2,24)_ = 0.987, *p* = 0.387; Figure 3C). A *post hoc* analysis showed that CO-rich saline increased the time spent in the center (*p* = 0.034). The one-way ANOVA on the NSF data revealed that CO-rich saline had a significant effect on latency to feeding (F_(2,28)_ = 8.892, *p* = 0.001; Figure 3D) without influencing the total intake (F_(2,28)_ = 2.391, *p* = 0.11; Figure 3E). The *post hoc* analysis showed that both 0.1 ml (*p* = 0.001) and 0.3 ml (*p* = 0.003) of CO-rich saline significantly decreased the latency to feeding. In the FST, CO-rich saline treatment had a significant effect on floating time (F_(2,20)_ = 4.274, *p* = 0.030; Figure 3F) without influencing the latency to floating (F_(2,20)_ = 2.817, *p* = 0.084; Figure 3G). The *post hoc* analysis showed that both 0.1 ml (*p* = 0.049) and 0.3 ml (*p* = 0.030) of CO-rich saline significantly decreased floating time. These behavioral results suggest that CO-rich saline produces rapid antidepressant- and anxiolytic-like effects.

### 3.5 CO Gas Pretreatment Exerted Significant Antidepressant- and Anxiolytic-Like Activities

**FIGURE 4:**
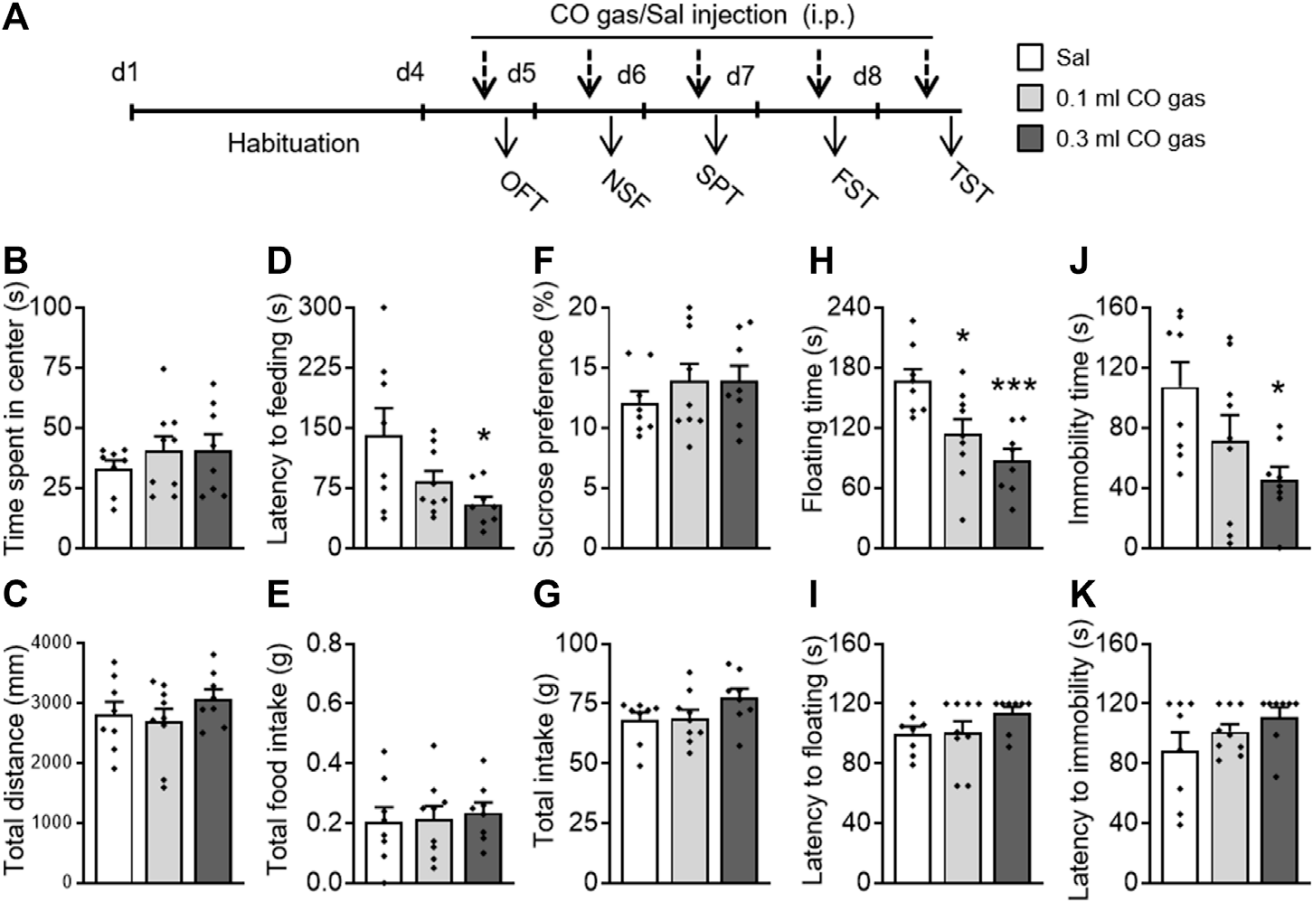
Systemic CO gas administration exerted rapid antidepressant- and anxiolytic-like activities in mice. **(A)** After a 3-day-adaptation, each mouse was treated with saline (0.3 ml, i.p.) or CO gas (0.1, 0.3 ml, i.p.) daily 30 min prior to behavioral test. Time spent in central zone **(B)** and total distance traveled **(C)** had no effects when treated with CO gas in the OFT. In the NSF test, latency to feeding decreased **(D)** but not total food intake **(E)**. No alteration occurred in sucrose preference **(F)** and in total intake **(G)** between three groups in the SPT. CO gas treatment decreased floating time **(H)** and immobility time **(J)**, but not floating latency **(I)** in the FST and the immobility latency **(K)** in the TST. The data are expressed as mean ± SEM and were analyzed with separate one-way ANOVAs. *p < 0.05, **p < 0.01, ***p < 0.001 as compared to the saline-treated group. OFT, open field test; NSF, novelty-suppressed feeding; SPT, sucrose preference test; FST, forced swimming test; TST, tail suspension test (*n* = 8–9 per group).

To investigate whether exogenous CO gas could also exert antidepressant- and anxiolytic-like effects, mice were administered with saline (0.3 ml, i.p.) or CO gas (0.1, 0.3 ml, i.p.) 30 min before behavioral testing (Figure 4A). There was no significant effect of CO gas on time spent in the center (F_(2,24)_ = 0.491, *p* = 0.618; Figure 4B) and total distance traveled (F_(2,24)_ = 1.07, *p* = 0.359; Figure 4C) in the OFT. A one-way ANOVA on the NSF data showed that CO gas had a significant effect on latency to feeding (F_(2,24)_ = 4.282, *p* = 0.027; Figure 4D) without influencing the total intake (F_(2,24)_ = 0.124, *p* = 0.884; Figure 4E). A *post hoc* analysis showed that 0.3 ml CO gas injection significantly decreased mice’s latency to feeding (*p* = 0.017). In the SPT, CO gas injection had no effect on sucrose preference (F_(2,24)_ = 2.294, *p* = 0.123; Figure 4F) and total intake (F_(2,24)_ = 0.772, *p* = 0.473; Figure 4G). In the FST, CO gas treatment had a significant effect on floating time (F_(2,24)_ = 9.051, *p* = 0.001; Figure 4H) without influencing the latency to floating (F_(2,24)_ = 1.615, *p* = 0.22; Figure 4I). The *post hoc* analysis showed that both 0.1 ml (*p* = 0.017) and 0.3 ml (*p* = 0.001) CO gas administration significantly reduced the floating time. CO gas treatment had a significant effect on immobility time (F_(2,24)_ = 4.145, *p* = 0.030; Figure 4J) without influencing the latency to immobility (F_(2,24)_ = 1.487, *p* = 0.246; Figure 4K) in the TST. The *post hoc* analysis showed that 0.3 ml CO gas administration significantly reduced floating time (*p* = 0.017). These results suggest that treatment with CO gas can produce significant antidepressant- and anxiolytic-like effects in mice.

### 3.6 CO Gas Pretreatment Significantly Blocked LPS-Induced Depressive- and Anxiety-like Behaviors

**FIGURE 5:**
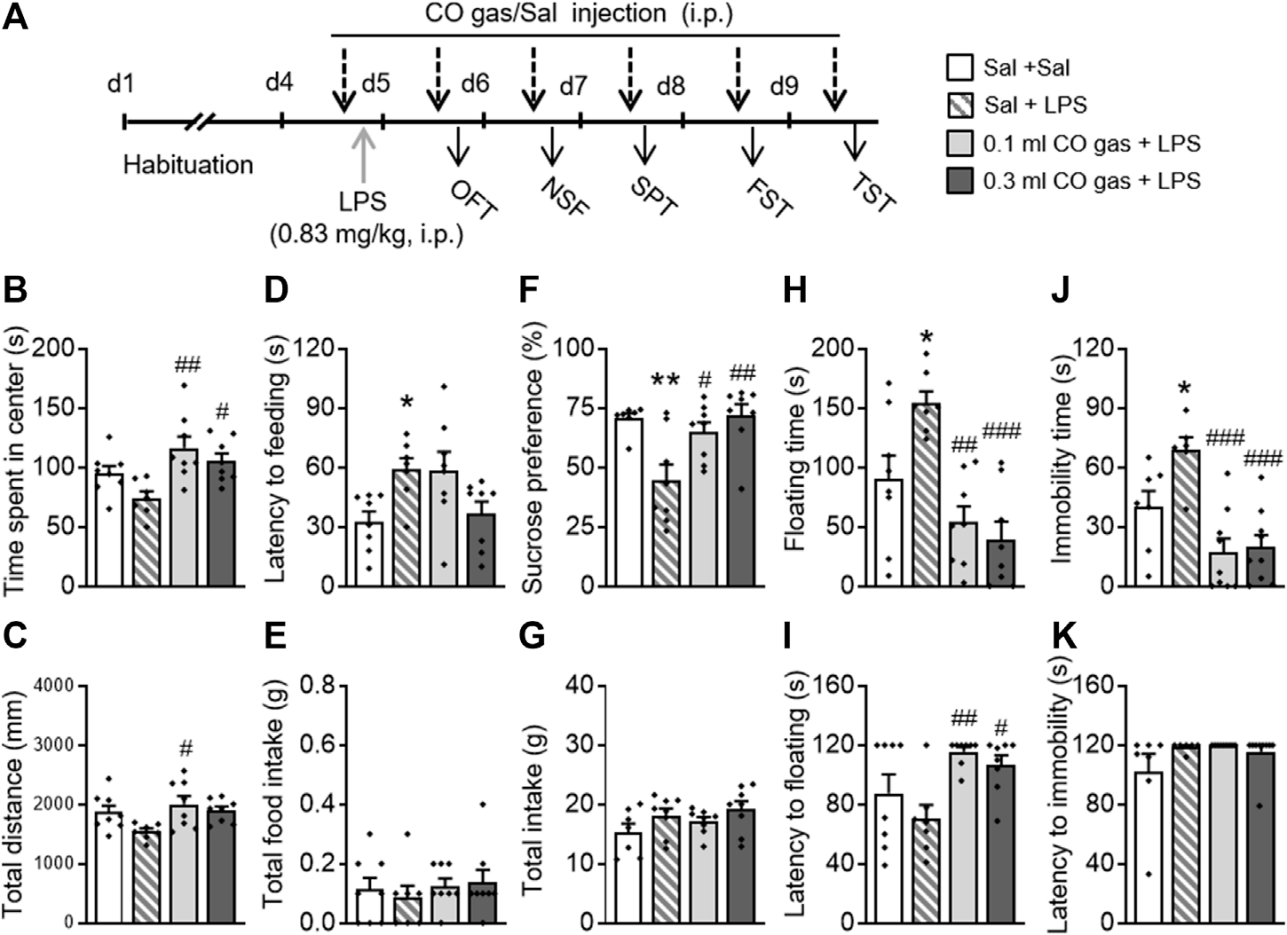
Systemic CO gas administration significantly blocked LPS-induced depressive and anxiety-like behaviors. **(A)** LPS (0.83 mg/kg, 0.2 ml/mouse, i.p.) was administered on the last day of the 3-day-adaptation period, while CO gas (0.1, 0.3 ml, i.p.) was administered 30 min before LPS injection and each behavioral test. CO gas treatment increased time spent in the center **(B)** and total distance **(C)** as compared to the LPS injection group in the OFT. In the NSF test, LPS treatment increased feeding latency **(D)** but not total food intake **(E)**. CO gas treatment significantly reversed the depressant-like state induced by LPS, reflected by the increased sucrose preference **(F)** but no change in total water intake **(G)** in the SPT. Increased floating time reversed by CO gas **(H)** and decreased floating latency **(I)** induced by LPS in the FST. In the TST, CO gas treatment significantly reversed the increased immobility time **(J)** induced by LPS but not latency to immobility **(K)**. The data are expressed as mean ± SEM and were analyzed with separate one-way ANOVAs. *p < 0.05, **p < 0.001 as compared to the Sal + Sal group; ^#^ *p* < 0.05, ^##^ *p* < 0.01, ^###^ *p* < 0.001 as compared to the Sal + LPS group; Sal, saline. LPS, lipopolysaccharide; OFT, open field test; NSF, novelty-suppressed feeding; SPT, sucrose preference test; FST, forced swimming test; TST, tail suspension test (*n* = 8–9 per group).

To provide additional evidence for the antidepressant- and anxiolytic-like effects of CO, a LPS-induced depression mice model was used (Figure 5A). The mice were randomly divided into four groups: 1) Sal + Sal group, untreated group neither with LPS or CO gas; 2) Sal + LPS group, treated with LPS once and 0.1 ml saline injection daily; 3) CO gas + LPS group, treated with LPS once and 0.1 ml CO gas injection daily; and 4) 0.3 ml CO gas + LPS group, treated with LPS once and 0.3 ml CO gas injection daily. LPS (0.83 mg/kg, 0.2 ml, i.p.) was administered for 3 days. After twenty-four hours, saline and CO gas (0.1 ml, 0.3 ml, i.p.) were administered 30 min before each behavioral experiment.

One-way ANOVAs on the OFT data revealed that there were differences in time spent in the center (F_(3,30)_ = 5.653, *p* = 0.004; Figure 5B) and total distance (F_(3,30)_ = 3.513, *p* = 0.029; Figure 5C) between groups. A *post hoc* analysis showed that both 0.1 ml (*p* = 0.001) and 0.3 ml (*p* = 0.018) CO gas treatments significantly increased time spent in the center, and that 0.1 ml CO gas treatment significantly increased total distance traveled (*p* = 0.012), as compared to the Sal + LPS group. In the NSF test, there was a significant difference in the latency to feeding between three groups (F_(3,30)_ = 4.080, *p* = 0.016; Figure 5D), while no difference was observed in total food intake (F_(3,30)_ = 0.094, *p* = 0.963; Figure 5E). *Post hoc* analyses showed that LPS prolonged the latency to feeding of mice (*p* = 0.036), which was not reversed by the CO gas treatment. In the SPT, there was a significant difference in the sucrose preference between groups (F_(3,30)_ = 4.080, *p* = 0.016; Figure 5F), while no difference was observed in total intake (F_(3,30)_ = 1.761, *p* = 0.176; Figure 5G). *Post hoc* analyses showed that LPS injection led to a significant decrease in sucrose preference (*p* = 0.003), which was reversed by 0.1 (*p* = 0.016) or 0.3 ml (*p* = 0.001) CO gas treatment. One-way ANOVAs on the FST data revealed that there were significant differences in floating time (F_(3,30)_ = 10.610, *p* < 0.001; Figure 5H) and latency to floating (F_(3,30)_ = 5.314, *p* = 0.005; Figure 5I). *Post hoc* analyses showed that LPS significantly prolonged the floating time (*p* = 0.019), which was reversed by 0.1 ml (*p* < 0.001) or 0.3 ml (*p* < 0.001) CO gas treatment, and that both 0.1 ml (*p* = 0.003) and 0.3 ml (*p* = 0.018) CO gas treatment increased the latency to floating, as compared to the Sal + LPS group. One-way ANOVAs on the TST data showed that there were significant differences in immobility time (F_(3,30)_ = 10.330, *p* < 0.001; Figure 5J) between groups, while no differences were observed in latency to immobility (F_(3,30)_ = 1.603, *p* = 0.209; Figure 5K). *Post hoc* analyses showed that LPS caused prolongation of immobility time (*p* = 0.034), which was reversed by 0.1 ml (*p* < 0.001) or 0.3 ml (*p* < 0.001) CO gas treatment.

These data suggest that CO gas reverses LPS-induced and anxiety-like behaviors in mice.

## 4 Discussion

In this study, we investigated the relationship between the HO-1/CO system and depression using the multifaceted approach. We found the following: 1) chemogenetic overexpression of HO-1 in the dorsal hippocampus produces, to a large extent, antidepressant-like effects in mice; 2) CO-RM-3 and CO-RM-A1 pretreatments exert cytoprotective effects against H_2_O_2_-induced cellular injury, and additionally RM-3 reverses the associated behavioral deficits; 3) CO-rich saline or CO gas administration produces antidepressant- and anxiolytic-like effects; and 4) CO gas has the ability to block LPS-induced behavioral deficits, as demonstrated in a number of behavioral paradigms such as the SPT, NSF, OFT, FST, and TST. Together, these findings suggest that the HO-1/CO system plays a critical role in the emotional state of the animals, and CO produces the antidepressant-like and anxiolytic effects, making the HO-1/CO system a potential pharmacotherapeutic target in the treatment of depression.

The HO-1/CO system has recently seen an explosion of research interest due to its neuroprotective, anti-inflammatory, and antioxidant effects (Jazwa and Cuadrado, 2010; Ryter and Choi, 2016; Lan et al., 2017). A number of studies have revealed that HO-1 has therapeutic potential under a variety of pathological conditions, including spinal cord injury, Alzheimer’s disease, Parkinson’s disease, and ischemic brain injury (Clark et al., 2003; Motterlini et al., 2005; Morroni et al., 2018; Zheng et al., 2019). Recent evidence suggests that the peripheral HO system is associated with the neuroprotective effects against LPS-induced behavioral abnormalities (Yin et al., 2019). Studies from our group as well as those of others reported that HO-1 might be associated with the pathological processes of depression and have antidepressant-like effects (Hodes et al., 2015; Yin et al., 2019). But until now, the precise role of the HO-1/CO system in depression has not been determined directly.

In this study, we utilized a multifaceted approach involving viral HO-1 overexpression in the hippocampus, CO-RM administration in the H_2_O_2_-induced cellular injury model, CO-rich saline, or CO gas administration alone or in combination with the LPS model of depression to systematically dissect the therapeutic and neuroprotective role of the HO-1/CO system in depression. In Exp. 1, we found that HO-1 produces antidepressant-like effects by using AAV-mediated overexpression of HO-1 in the dorsal hippocampus. Mice with overexpression of hippocampal HO-1 spent a significantly shorter amount of time floating in the FST and being immobile in the TST than the naïve or AAV-GFP–treated mice, suggesting that the HO-1/CO system plays a critical role in the emotional state of the animals. Surprisingly, overexpression of HO-1 did not affect sucrose preference. One possible explanation of these negative results might be that the SPT extrapolates the hedonic aspect of depression, and the FST and TST focus on learned helplessness commonly associated with depression. Thus, it is possible that the hippocampal HO-1/CO system is involved in different aspects of the emotional state of the animal and different depression-like symptoms. Clearly, more research is needed to dissect the function of the HO-1/CO system in the dorsal hippocampus.

Given that the activation of this system led to a reduction in depressive-like symptoms and that the HO-1/CO system was involved in the emotional state of the animals, we then investigated (Exp. 2) whether the CO-RM administration can also reduce depressive-like symptoms and provide neuroprotection against H_2_O_2_-induced cellular injury. The results of *in vitro* experiments with the H_2_O_2_-induced cellular injury model (Exp. 2) showed that CO-RM-3 and CO-RM-A1 pretreatments exert cytoprotective effects. Specifically, cultures with H_2_O_2_ cellular injury showed a higher cell survival ratio when pretreated with CO-RMs, suggesting that the CO-RM treatment can have a protective or prophylactic function.

This parallels with previous reports that CO prevents neuronal apoptosis, improves neuronal differentiation, and stimulates endogenous neurogenesis (Almeida et al., 2016a; Choi et al., 2016; Dreyer-Andersen et al., 2018). Moreover, in traumatic brain injury models, CO has been found to promote nerve regeneration and functional recovery by preventing pericyte death and strengthening a cross talk between the pericytes and neural stem cells to help them differentiate into mature neurons (Almeida et al., 2016b; Choi et al., 2016). These effects might be in part mediated by CO-induced phosphorylation of nitric oxide synthase (Anagnostaras et al., 1999), leading to the increase of neurogenesis, NeuN, and BrdU-positive cells that promote nerve degeneration and functional recovery. Our study is in line with previous reports and provides evidence that CO exerts the cytoprotective effects against H_2_O_2_-induced oxidative injury of PC12 cells, decreasing the apoptosis of cells and associated behavioral deficits (see *Discussion* below)*.*

CO, an endogenous product of heme degradation, is known for its toxic effects at high levels but for its cytoprotective and neuroprotective effects at lower levels (Verma et al., 1993; Herman, 1997; Queiroga et al., 2015). Like other gaseous molecules (e.g., hydrogen sulfide and sulfur dioxide) that exert rapid antidepressant- and anxiolytic-like effects through anti-inflammatory and antioxidative mechanisms in rodents (Liu et al., 2017a; Hou et al., 2017; Shi et al., 2020), endogenous and/or exogenous CO produces significant regulatory effects through the same mechanisms and also exerts rapid protective effects in the central nervous system (Queiroga et al., 2015). Moreover, there is evidence that CO administration facilitates the induction of hippocampal long-term potentiation, augments synaptic neurotransmission, and is involved in the regulation of synaptic plasticity (Verma et al., 1993; Herman, 1997; Wang et al., 2018a), all of which are associated with many neuropsychiatric conditions, including depression and anxiety. The neuroprotective effects of CO gas have been recapitulated by CO-RMs as demonstrated by previous studies showing that 1) CO-RM-3 attenuates inflammation induced by LPS and other inflammatory markers in microglia cells (Bani-Hani et al., 2006), 2) CO-RM-3 reduces brain damage and inflammation in a model of hemorrhagic stroke (Yabluchanskiy et al., 2012), and 3) CO-RM-A1 displays cerebroprotective effects against vascular dysfunction and apoptosis in different models of brain-induced injury (Basuroy et al., 2006; Basuroy et al., 2013).

Here, using different models and approaches, we demonstrated that CO administration produces antidepressant effects. As mentioned previously, we found that CO-RM-A1 and CO-RM-3 exert cytoprotective properties, but to our surprise, CO-RM-A1 did not produce antidepressant- and anxiolytic-like effects (Exp. 2). In contrast, CO-RM-3 significantly reduced depressive-like symptoms, as indicated by a reduction in floating time and increased latency to floating in the FST. These findings suggest that CO-RM-3 might have a unique target involved in the emotional state.

In Exp. 3 and 4, we demonstrated that CO-rich saline or CO gas produces antidepressant- and anxiolytic-like effects in mice. Specifically, in the OFT, mice pretreated with CO-rich saline (Exp. 3) spent more time in the center of the open field than the control group. In the NSF, CO-treated mice more readily reached for food, as demonstrated by shorter latencies to feeding and spent less time floating in the FST, than the control. Similar findings were obtained when mice were treated with CO gas (Exp. 4). Significant behavioral improvements were observed in CO gas–treated mice in the NSF, FST, and TST but not OFT and SPT. These behavioral results suggest that CO-rich saline and CO gas produce rapid antidepressant effects and both should be explored as potential therapeutics for the treatment of depression.

Last, using CO gas injection, we demonstrated the rapid-acting effects of CO on LPS-induced behavioral deficits, suggesting that CO at small doses has therapeutic potential. Our multifaceted approach involving chemogenetic overexpression of hippocampal HO-1, pharmacological administration of CO-rich saline, or CO gas in the absence or presence of LPS administration–blunted depression- and anxiety-like symptoms, as demonstrated in the battery of behavioral tests such as SPT, FST, TST, and OFT, and suggests that the HO-1/CO system might be a new therapeutic target in the treatment of depression.

It is worth noting that in our study overexpression of HO-1 that leads to CO-release produced only partial antidepressant effects (Exp. 1) as opposed to more robust effects observed with the pharmacological manipulations (Exp. 2–5). These discrepancies in the results might be in part due to differences in the manipulation of the CO/HO-1 system. Chemogenetic overexpression of HO-1 occurred on a local, hippocampal level, while CO systemic administration targeted the central CO-HO-1 system. Another discrepancy in results pertains to the fact that CO gas produced the anxiolytic-like effect in the NSF test in naïve rats but not in the LPS-treated mice (Exp. 5). One possible explanation for this discrepancy is that a much higher dose of CO is most likely needed to block LPS deficits.

To control for false-positive findings and avoid a type 1 error, we examined closely the locomotor activity from the open field test. Mice with chemogenetic overexpression of HO-1 (Exp. 1) or those subjected to the CO-RM treatment (Exp. 2), CO-rich saline (Exp. 3), or CO gas treatment in the absence (Exp. 4) or presence of LPS administration (Exp. 5) showed the same level of locomotor activity, measured as total distance traveled, as their relevant controls. Thus, reductions in behavioral parameters in the NFT, FST, and TST cannot be explained as motoric impairment but rather as reductions in depressive-like symptoms caused by overexpression of HO-1, CO-RM administration, or CO administration.

Although the precise CO mechanisms are not fully known, they appear to resemble the actions of commonly prescribed antidepressants (e.g., selective serotonin reuptake inhibitors) that reduce antidepressant symptoms by activating the monoamine systems and stimulating neurogenesis in the hippocampus (Santarelli et al., 2003). We indeed observed that the hippocampal HO-1/CO system is involved in the depressive-like state of the animals, and CO-RM-3 administration can reverse the depressive-like symptoms. CO is known for its neuroprotective effects in the cortical amygdala and hippocampal neurons (Hettiarachchi et al., 2014; Wang et al., 2017; Riego et al., 2018); the brain regions are involved in depression (Sheline et al., 2002; Whalen et al., 2002; Liu et al., 2017b; Seo et al., 2017; Du Preez et al., 2021; Garro-Martinez et al., 2021; Li et al., 2021; Ouyang et al., 2021; Qin et al., 2021; Sindermann et al., 2021).

Thus, it is conceivable that the observed depressant-like symptoms are mediated by the cortico-limbic circuitry, and neuroprotective and antidepressant effects of CO involve mechanisms within this circuitry. One possibility is that the CO/HO-1 system influences the expression of trophic factors in the amygdala, hippocampus, and frontal cortex, contributing to the improvement of the emotional state of the animal. Indeed, neurotrophic factors (especially brain-derived neurotrophic factor (BDNF)) and neurogenesis have been implicated in the action of antidepressants (Santarelli et al., 2003) and CO (Almeida et al., 2016a), both of which target the monoaminergic systems within the cortico-limbic circuitry (Taskiran et al., 2003). Although this is speculative at this point, such hypotheses raise interesting questions and warrant further investigation.

Future studies should also investigate whether the similar therapeutic and neuroprotective effects of CO can be observed in females. This is especially important, given the fact that depression is more common in females and males. Because this study involved the multifaceted approach and multiple experiments, we decided to focus only on males, but we note that this is a severe limitation of our study. We acknowledge the importance of future studies with female subjects to ensure reproducibility and generalization of our findings and to investigate potential therapeutics for women struggling with depression. Indeed, the major depressive disorder has one of the highest prevalence among mental illnesses (Trivedi et al., 2006; Kaufman, 2018; Domschke and Gottschalk, 2019), affecting millions of individuals around the world each year. Thus, the search for rapid and effective antidepressants remains a high priority in the scientific community, and this study provides a unique insight into mechanisms underlying depression and proposes a novel therapeutic approach.

In conclusion, the present study supports the hypothesis that the gaseous molecule CO administration exerts a regulatory role in depression. We directly confirmed that CO supplement by both endogenous HO-1 overexpression in the dorsal hippocampus and exogenous CO treatment (including CO-RM, CO gas, and/or CO-rich saline injection, i.p.) exerts significantly rapid antidepressant- and anxiolytic-like effects. These findings extend our understanding of neuroprotective functions of gaseous molecule CO and highlight novel targets for the development of rapid-acting therapeutics for the treatment of depression.

## Data Availability

The raw data supporting the conclusion of this article will be made available by the authors, without undue reservation.
